# Urinary Bladder Acute Inflammations and Nephritis of the Renal Pelvis: Diagnosis Using Fine-Tuned Large Language Models

**DOI:** 10.3390/jpm15020045

**Published:** 2025-01-24

**Authors:** Mohammad Khaleel Sallam Ma’aitah, Abdulkader Helwan, Abdelrahman Radwan

**Affiliations:** 1 Electrical Engineering/Robotics and Artificial Intelligence Engineering, Faculty of Engineering & Technology, Applied Science Private University, Amman 11931, Jordan; a_radwan@asu.edu.jo; 2 Department of Health, Medicine and Caring Sciences, Linköping University, 581 85 Linköping, Sweden

**Keywords:** large language models, LLMs, NLP, autoregressive, transformer, GPT-2, BERT, Distill-BERT, TinyBERT, supervised fine-tuning, SFT

## Abstract

**Background:** Large language models (LLMs) have seen a significant boost recently in the field of natural language processing (NLP) due to their capabilities in analyzing words. These autoregressive models prove robust in classification tasks where texts need to be analyzed and classified. **Objectives:** In this paper, we explore the power of base LLMs such as Generative Pre-trained Transformer 2 (GPT-2), Bidirectional Encoder Representations from Transformers (BERT), Distill-BERT, and TinyBERT in diagnosing acute inflammations of the urinary bladder and nephritis of the renal pelvis. **Materials and Methods:** the LLMs were trained and tested using supervised fine-tuning (SFT) on a dataset of 120 examples that include symptoms that may indicate the occurrence of these two conditions. **Results:** By employing a supervised fine-tuning method and carefully crafted prompts to present the data, we demonstrate the feasibility of using minimal training data to achieve a reasonable diagnostic, with overall testing accuracies of 100%, 100%, 94%, and 79%, for GPT-2, BERT, Distill-BERT, and TinyBERT, respectively.

## 1. Introduction

Acute inflammation of the urinary bladder, known as acute cystitis, is an abrupt and frequently painful condition marked by the inflammation of the bladder’s lining [1]. This condition is predominantly triggered by bacterial infections, with *Escherichia coli* being the most common pathogen implicated in these cases. Acute cystitis represents a specific form of urinary tract infection (UTI) that targets the bladder, resulting in a variety of discomforting symptoms [1,2].

Acute inflammation of the urinary bladder is marked by the abrupt onset of abdominal pain and a persistent urge to urinate, accompanied by painful micturition and, in some cases, difficulty in retaining urine [2]. This disease is characterized by an elevated body temperature, typically not exceeding 38 °C. The urine produced is often cloudy and may contain blood. With appropriate treatment, symptoms generally subside within a few days; however, there is a tendency for recurrence. Individuals experiencing acute urinary bladder inflammation may be at risk of the condition developing into a chronic form [3].

Acute nephritis of the renal pelvis, commonly known as acute pyelonephritis, is a critical medical issue marked by the inflammation of both the renal pelvis and the surrounding kidney tissue, predominantly resulting from a bacterial infection [4]. This condition frequently develops as a consequence of an ascending urinary tract infection (UTI), wherein bacteria migrate from the bladder through the ureters to the kidneys.

This condition, originating from the renal pelvis, is significantly more prevalent in women than in men. The condition typically presents with a sudden onset of fever, which can reach or even surpass 40 °C [1]. This fever is often accompanied by chills and unilateral or bilateral lumbar pain, which can be quite severe [1,5]. Symptoms indicative of acute inflammation of the urinary bladder frequently manifest as well. Additionally, it is not uncommon for patients to experience nausea, vomiting, and diffuse abdominal pain [5,6].

Conventional diagnostic methods of such conditions, although effective, often require considerable time and may fail to fully address the complexities of patient presentations. In this regard, utilizing advanced large language models (LLMs) such as BERT [7] and GPT-2 [8] presents a valuable opportunity to improve both the accuracy and efficiency of diagnostics.

The utilization of large language models (LLMs) in the field of healthcare has significantly transformed numerous facets of medical practice, especially in the area of diagnostics [9]. These models are proficient in analyzing extensive volumes of textual information, which encompasses patient histories, clinical documentation, and laboratory findings. By tailoring these models to numerical datasets related to urinary tract disorders, we can improve their capacity to detect patterns and relationships that may not be readily observable to human practitioners [10]. For example, research has indicated that LLMs can aid in forecasting disease outcomes based on symptom descriptions and clinical information [8,9]. This functionality is particularly vital in the diagnosis of conditions such as acute nephritic syndrome, where symptoms like hematuria and reduced urine output necessitate meticulous interpretation [11,12].

The fine-tuning process of these LLMs further customizes these models to align with the particular language and context associated with medical diagnostics [2,10,13]. By training large language models (LLMs) on datasets that encapsulate the clinical language relevant to urinary bladder inflammations and renal pelvis nephritis, we can enhance their predictive accuracy. This level of specificity is crucial, as it enables the models to grasp the nuances of medical terminology and patient presentations, resulting in more dependable diagnostic outputs. Incorporating LLMs into clinical workflows has the potential to substantially decrease diagnostic errors and improve patient care by equipping clinicians with effective decision-support tools.

In this work, GPT-2 [8], BERT [7], Distill-BERT [14], and TinyBERT [15] were fine-tuned on a classification-based dataset [16], which comprises medical attributes that can indicate the occurrence of acute inflammations of the urinary bladder and nephritis of the renal pelvis. After fine-tuning, the textual analysis capability of these LLMs can help analyze the symptoms and decide whether an instance represents a potential patient or not. Table 1 show the exact models employed in this study, in addition to their sizes and descriptions.

The rest of this paper is organized as follows: Section 2 is a review of the related studies and papers. Section 3 gives the materials and methods, which include a dataset description, prompt creation, and the fine-tuning process of the models. Section 4 presents the results, and Section 5 offers a discussion of the results. Finally, Section 6 sets out the conclusion of this work.

## 2. Review

The employment of machine learning (ML) models, particularly large language models (LLMs), in diagnosing acute urinary bladder inflammation and nephritis has attracted considerable interest in contemporary research. This review consolidates the results from multiple studies that have utilized these computational methods to improve diagnostic precision and a predictive performance in urological disorders.

A recent study [17] introduced three distinct machine learning models—logistic regression, decision tree, and random forest—aimed at predicting recurrent urinary tract infections (RUTIs) attributed to *Escherichia coli*. Among these, the random forest model exhibited the greatest accuracy in predictions, underscoring the potential of machine learning to proficiently evaluate both host and bacterial traits in predicting RUTIs. Furthermore, the decision tree model showed significant classification accuracy within particular patient subgroups, indicating that customized strategies may enhance clinical outcomes for vulnerable populations.

A separate investigation [3] assessing GPT-4’s role as a diagnostic support tool revealed that, although it was capable of producing differential diagnoses with a satisfactory level of accuracy, it encountered difficulties when faced with intricate cases. The research underscored the promise of large language models (LLMs) in aiding healthcare professionals by offering a list of potential diagnoses; however, it emphasized that these models should not supplant human judgment. This finding carries significant implications for the incorporation of LLMs into clinical settings, especially in complex diagnostic situations.

Moreover, a research study concentrated on uncomplicated urinary tract infections employed a range of artificial intelligence methodologies, such as decision trees and artificial neural networks (ANNs), to assess the probability of conditions like cystitis [18]. The ANN model attained a remarkable accuracy of 98.3%, highlighting the potential of machine learning techniques in the diagnosis of urinary disorders by analyzing clinical symptoms alongside laboratory findings.

Another study examined the application of machine learning techniques to predict damage to the upper urinary tract by integrating inflammatory markers with conventional clinical indicators. This methodology demonstrated the capacity of machine learning to improve early detection and risk assessment in individuals at risk of urinary tract injury, emphasizing its significance in the realm of preventive healthcare [19].

## 3. Materials and Methods

### 3.1. Dataset Description

The Acute Inflammations dataset, obtained from the UCI Machine Learning Repository [16], is a publicly available dataset that was curated by Dr. Jacek Czerniak of the Systems Research Institute, Polish Academy of Sciences, Laboratory of Intelligent Systems in Warsaw, Poland. This dataset comprises 120 instances, each characterized by six attributes, as shown in Table 2.

**Figure 1:**
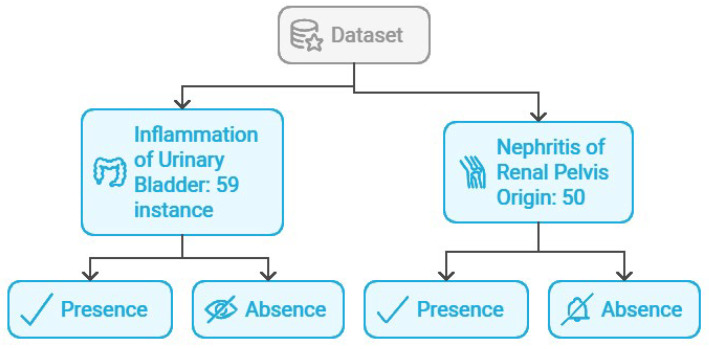
Acute Inflammations dataset [11]. The dataset comprises 120 instances in total; amongst them, 59 have bladder inflammation and 50 have nephritis of the renal pelvis. The remaining 11 instances have none of these conditions. The presence of a condition was noted as ‘Yes’, while the absence of it was noted as ‘No’.

The dataset presents two binary class labels, as shown in Figure 1:

- Inflammation of urinary bladder: Indicates the presence or absence of urinary bladder inflammation.
- Nephritis of renal pelvis origin: Indicates the presence or absence of nephritis originating in the renal pelvis.

Of the 120 instances, 59 exhibit inflammation of the urinary bladder, while 50 exhibit nephritis of renal pelvis origin.

### 3.2. Data Preprocessing and Prompt Engineering

Recently, large language models have made a significant breakthrough in the domain of natural language processing (NLP) and have attracted substantial attention [14,19]. These autoregressive models are characterized by their huge parameter counts, extensive pre-training on large datasets of text, and subsequent fine-tuning for targeted applications [14,15,17].

The method used for fine-tuning the large language models (LLMs) in this work is referred to as supervised fine-tuning (SFT) [20], which involves adapting a pre-trained model, such as GPT-2, to a specific downstream task using labeled data. In this case, LLMs such as GPT-2 should be fine-tuned to diagnose the acute inflammations of the urinary bladder and nephritis. However, this process involves several steps such as dataset preparation and prompt engineering.

#### 3.2.1. Dataset Preparation

Dataset preparation is the process where we prepare our dataset so that an LLM can understand it. As shown in Table 1, some parameters are categorical (Yes, No) while the temperature parameter is numerical (35.5–41.5 °C), and since LLMs are language models, we kept the categorical parameters as they were, whereas the temperature feature was normalized using scikit-learn’s StandardScaler to ensure all features were on a comparable scale.

The next step was to encode the labels, in which the output labels for bladder inflammation and nephritis were encoded as binary values (1 for “yes” and 0 for “no”). This transformation converted the LLMs into classifications models.

The last step was to split the data into training (60%) and testing (40%) sets using a train–test split function with a fixed random state for reproducibility.

#### 3.2.2. Prompt Engineering

Once the dataset [16] is prepared and processed, it is time to create the prompts that will be used to train the models. LLMs are language models, i.e., they understand language, unlike classical image classification models, which work with images or numerical values only. Thus, we engineered prompts to structure the input data in a prompt-completion format suitable for training the language model as shown in Box 1. The prompt template was designed as follows:

Box 1 **Prompt engineering of the input data.**

{“prompt”: “Diagnose urinary tract conditions based on the following symptoms:\nTemperature: −0.72\nNausea: No\nLumbar Pain: No\nUrine Pushing: No\nMicturition Pains: No\nBurning of Urethra: No\n\nDiagnosis:”, “completion”: “Bladder inflammation: No\nNephritis: No”}

This prompt structure was crafted to mimic the clinical presentation of symptoms, allowing the model to interpret the input as a diagnostic classification task.

### 3.3. Supervised Fine-Tuning of the LLMs

To optimize the LLMs’ performance while addressing computational constraints, we selected pre-trained LLMs that are relatively small in size for the task of acute inflammation and nephritis diagnosis. For instance, we selected the GPT-2 base model, which has 124 million parameters, BERT base model, which has 110 million parameters, Distill-BERT, which has approximately 66 million parameters, and TinyBERT, which has approximately 14.5 million parameters.

The four employed models—GPT-2, BERT, Distill-BERT, and TinyBERT—utilize different architectural frameworks and pre-training goals. GPT-2 is based on a decoder-only transformer architecture, whereas the BERT variants are structured around an encoder-only framework.

**Figure 2:**
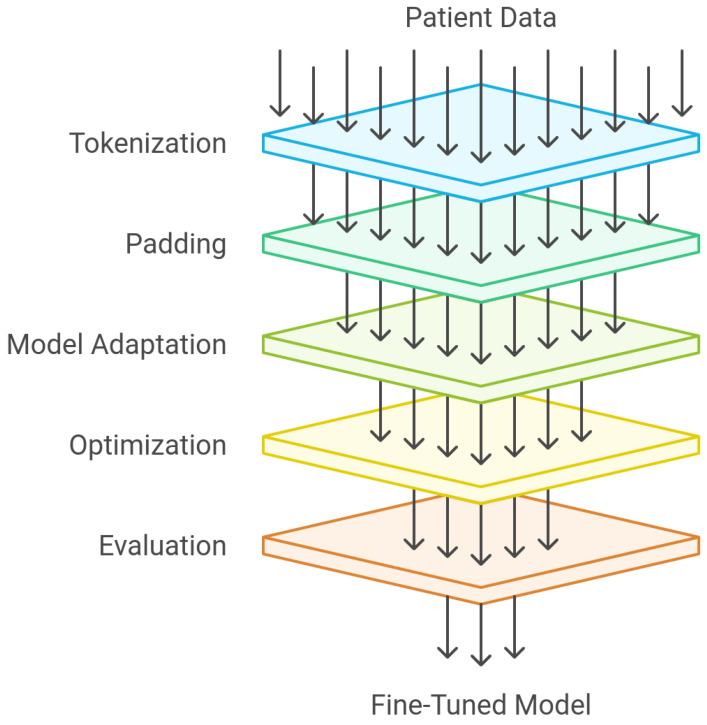
The fine-tuning process of the LLMs.

Before fine-tuning, the text data, comprising patient symptoms and diagnoses, were tokenized using the GPT-2 tokenizer, in the case of GPT-2. This process involves breaking down text into smaller units called tokens. The tokenizer of every LLM employs a subword tokenization technique that efficiently handles out-of-vocabulary words by breaking them into smaller subword units. This approach allows for a flexible vocabulary and better handling of unseen words. Once tokenized, the sequences were padded to a fixed length, ensuring a consistent input to the model. The resulting tokenized sequences, along with their corresponding labels, were fed into the fine-tuning process. Figure 2 shows the fine-tuning process of the LLMs. Table 3 shows the performance of each model.

The pre-trained LLMs were adapted to their new target task by modifying their output layers to have two neurons, corresponding to the two disease classes: bladder inflammation and nephritis. The AdamW optimizer with a learning rate of 2 × 10^−5^ was used to update the model’s parameters during training. For each training batch, the model generated predictions, calculated the cross-entropy loss, and updated its parameters through backpropagation. Each model was trained for 20 epochs, with evaluation on both training and test sets after each epoch to monitor the performance and prevent overfitting. In this study, all experiments were conducted using Python 3.9 with PyTorch (v2.0.0) and the Transformers library (v4.30.0) for loading and fine-tuning pre-trained models (GPT-2, BERT, Distill-BERT, TinyBERT). The models were implemented on an NVIDIA GTX 1650 Ti GPU using CUDA (v11.7) for acceleration. Data preprocessing, including tokenization and padding, was performed using the tokenizers provided by the Transformers library.

#### 3.3.1. Comparison of LLMs with Shallow Neural Networks

To explore the power of fine-tuning large language models (LLMs) compared to shallow neural networks, we trained two baseline models on our dataset:

- A three-layer feedforward neural network (FFNN): This model consisted of an input layer, a hidden layer with 128 units and ReLU activation, and an output layer with softmax activation for classification.
- A one-dimensional convolutional neural network (1D-CNN): This model included a 1D convolutional layer with 64 filters, a kernel size of 3, and ReLU activation, followed by a max-pooling layer and a fully connected layer for classification.

Both models were trained using the Adam optimizer with a learning rate of 1 × 10^−3^ and a batch size of 32. Early stopping was employed to prevent overfitting, and the models were evaluated on the same test set used for the LLMs. The results of this comparison are presented in Table 4, which shows the accuracy of the shallow neural networks versus the fine-tuned LLMs.

#### 3.3.2. Few-Shot Learning with DeepSeek Chat

For comparison purposes, we employed a few-shot learning approach to evaluate the performance of DeepSeek Chat [21,22], a cutting-edge large language model based on the GPT architecture with approximately 175 billion parameters. The specific version used was DeepSeek Chat v1.0, which is optimized for natural language understanding and generation tasks.

Fine-tuning involved further training pre-trained models (e.g., GPT-2, BERT) on a labeled dataset, updating their weights to learn task-specific patterns, and achieving a high performance at the cost of computational resources. In contrast, few-shot learning utilized DeepSeek Chat with only two examples to guide predictions, leveraging its pre-trained knowledge without weight updates, making it efficient for low-data scenarios but with a slightly lower accuracy.

To guide the model, we crafted a prompt that included two examples, one for each class, to establish the initial classification criteria. The prompt was designed to help the model understand the relationship between symptoms and diagnoses. The structure of the prompt was as follows:

Input:
Temperature: [value], Nausea: [yes/no], Lumbar pain: [yes/no], Urine pushing: [yes/no], Micturition pains: [yes/no], Burning urethra: [yes/no]
Output:
Inflammation of urinary bladder: [yes/no], Nephritis of renal pelvis origin: [yes/no]

## 4. Results

**Figure 3:**
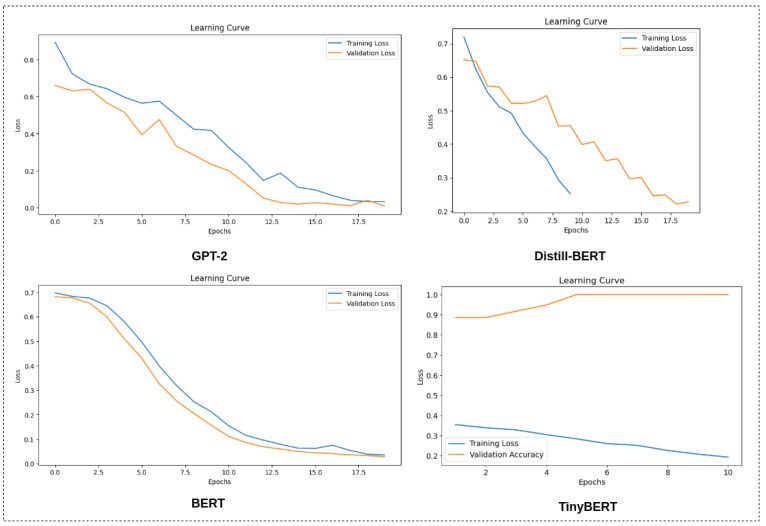
Learning curves of the models. Row 2 shows the learning curves of GPT-2 and Distill-BERT, while row 2 shows the learning curves of BERT and TinyBERT.

In this section, we report the results achieved by the LLMs during training and testing. We selected the Accuracy, Precision, F1-score, and Recall as the evaluation metrics of the models. Note that the models were tested on 40% of the data. Figure 3 shows the learning curves of the four different models in the training phase. Note that all models were trained on 60% of the data and for 20 epochs. Table 3 shows the performance evaluation metrics of the models when tested on 40% of the remaining data.

### 4.1. Impact of LLMs on the Diagnosis of Acute Inflammation and Nephritis

To explore the power of LLM fine-tuning over shallow neural networks, we trained a simple three-layer feedforward neural network and a one-dimensional convolutional neural network (1D-CNN) on our dataset to compare their performance to that of the LLMs.

Table 4 shows the comparative results of neural networks versus LLMs in terms of accuracy.

### 4.2. Few-Shot Learning of DeepSeek Model

For comparison purposes, we employed a few-shot learning approach to evaluate the performance of a cutting-edge large language model called DeepSeek Chat [21,22] for classifying patients with acute inflammation or nephritis into either “Inflammation of urinary bladder” or “Nephritis of renal pelvis origin” based on their clinical symptoms. We utilized a dataset comprising 120 records [16], each with features such as temperature, nausea, lumbar pain, urine pushing, micturition pains, and burning urethra. To guide the model, we crafted a prompt that included only two examples, one for each class, which were used to establish the initial classification criteria. The prompt was designed to guide the model in understanding the relationship between symptoms and the corresponding diagnoses. Specifically, the prompt had the following structure [16]:

Input: Temperature: [value], Nausea: [yes/no], Lumbar pain: [yes/no], Urine pushing: [yes/no], Micturition pains: [yes/no], Burning urethra: [yes/no]

Output: Inflammation of urinary bladder: [yes/no], Nephritis of renal pelvis origin: [yes/no]

For instance, the two examples provided were the following [16]:

1. Input: Temperature: 35.9, Nausea: no, Lumbar pain: no, Urine pushing: yes, Micturition pains: yes, Burning urethra: yes
  Output: Inflammation of urinary bladder: yes, Nephritis of renal pelvis origin: no
2. Input: Temperature: 40.0, Nausea: yes, Lumbar pain: yes, Urine pushing: yes, Micturition pains: yes, Burning urethra: yes
  Output: Inflammation of urinary bladder: no, Nephritis of renal pelvis origin: yes

These examples were used to prompt the DeepSeek Chat model [21], and its predictions were then compared against ground truth labels for the remaining 118 records [16] to assess its accuracy. Table 5 shows the results of the few-shot learning of the DeepSeek Chat model. The overall accuracy of this model was calculated to be 75%. Additionally, we computed the precision, recall, and F1-score for each class to provide a comprehensive evaluation. For “Inflammation of urinary bladder”, the model achieved a precision of 0.875, recall of 0.875, and F1-score of 0.875. Conversely, for “Nephritis of renal pelvis origin”, the precision was 0.5, recall was 0.25, and F1-score was 0.3333. These metrics highlight the model’s strong performance in identifying inflammation of the urinary bladder but indicate its room for improvement in diagnosing nephritis of renal pelvis origin, suggesting potential areas for further refinement and validation.

The model used in this few-shot learning comparative experiment was DeepSeek Chat, a large language model developed by DeepSeek [22]. It is based on the GPT architecture and has approximately 175 billion parameters. The specific version used was DeepSeek Chat v1.0, which is optimized for natural language understanding and generation tasks.

## 5. Discussion

This study aimed to explore the power of fine-tuned LLMs in acute inflammation and nephritis of renal pelvis diagnosis. Our research addresses the need to leverage LLMs in the medical field where data need to be retrieved and analyzed to make medical decisions. Large language models (LLMs) like GPT-2 and BERT have significant potential for medical diagnostic tasks, particularly those involving the classification of conditions such as acute inflammation and nephritis. The ability of LLMs to analyze and interpret complex linguistic patterns, coupled with their extensive training on diverse text corpora, makes them well-suited for tasks where the primary data consist of descriptive symptoms and clinical parameters. Unlike traditional neural networks, which may require extensive feature engineering and large datasets, LLMs can leverage their natural language processing capabilities to understand and classify symptoms directly from textual descriptions. This inherent advantage positions LLMs as a superior choice for such diagnostic tasks, offering a more intuitive and efficient approach to medical classification based on symptom-based datasets.

The models selected for this research were GPT-2, BERT, Distill-BERT, and TinyBERT. These models were trained and evaluated using the dataset [16]. Figure 3 illustrates the training loss for these models over 20 epochs. Notably, GPT-2 and BERT achieved excellent training loss (100%) without exhibiting signs of overfitting or hallucination. In contrast, Distill-BERT and TinyBERT did not attain such low training loss. The learning curve for TinyBERT suggests an initial high accuracy (~90%), which abruptly increased to 100%, potentially indicating overfitting.

This training performance was reflected in the test set results, as presented in Table 3. GPT-2 and BERT demonstrated superior diagnostic capabilities for acute inflammation and nephritis of the renal pelvis, achieving 100% for their accuracy, precision, F1-score, and recall. Conversely, Distill-BERT and TinyBERT exhibited lower performance metrics, with reduced scores across the accuracy, precision, F1-score, and recall.

An additional experiment was conducted to investigate the efficacy of large language models (LLMs) in diagnosing acute inflammation of the urinary bladder and nephritis of the renal pelvis by analyzing symptoms and interpreting complex linguistic patterns indicative of these conditions. In this experiment, a simple three-layer artificial neural network (ANN) and a convolutional neural network (1-CNN) were employed. Both networks were trained on the same dataset used for the LLMs. The results are summarized in Table 4. Notably, the LLMs demonstrated a superior performance in terms of accuracy compared to the ANN and 1-CNN. This superior performance highlights the potential of LLMs to revolutionize medical diagnostics, providing a more accurate and effective method for classifying diseases based on symptom-driven datasets.

Furthermore, Table 5 presents the results of an experiment involving an interactive instruction-based chat model (DeepSeek Chat) prompted using few-shot learning, to assess its ability to diagnose acute inflammation of the urinary bladder and nephritis using only two examples during training. The findings in Table 5 indicate that, despite having more parameters and being trained on larger datasets, DeepSeek Chat did not surpass any of the fine-tuned LLMs utilized in this study, such as GPT-2, BERT, Distill-BERT, and TinyBERT, which are smaller in size. This underscores the efficacy of supervised fine-tuning methods for LLMs, particularly when considering the differences in model size (number of parameters) and the size of the training corpus between the fine-tuned models and DeepSeek Chat.

Lastly, despite the impressive performance demonstrated by the LLMs fine-tuned using our dataset, these models exhibit certain limitations. The models were evaluated on a relatively small dataset, which restricts the generalizability of these findings from this specific study. A more extensive dataset, encompassing thousands of testing examples, would enhance the reliability and feasibility of these conclusions, providing a more robust validation of the LLMs’ diagnostic capabilities.

## 6. Conclusions

This study has explored the potential of large language models (LLMs) in diagnosing acute inflammation of the urinary bladder and nephritis of the renal pelvis using symptom-based datasets. By employing a supervised fine-tuning method and carefully crafted prompts to present the data, we demonstrated the feasibility of using minimal training data to achieve a reasonable diagnostic accuracy. The LLMs—GPT-2, BERT, Distill-BERT, and TinyBERT—were fine-tuned on a dataset of 120 records and achieved overall accuracies of 100%, 100%, 94%, and 79%, respectively.

Additionally, precision, recall, and F1-score metrics were computed for each class, highlighting the models’ strong performance in identifying inflammation of the urinary bladder but indicating their room for improvement in diagnosing nephritis of renal pelvis origin.

Despite the promising results, this study has limitations. The models were tested on a small dataset, which restricts the generalizability of these findings. A larger dataset with thousands of testing examples will enhance the reliability and feasibility of these conclusions, providing a more robust validation of the LLMs’ diagnostic capabilities.

In conclusion, this paper contributes to the field by demonstrating the potential of fine-tuning LLMs in medical diagnostics, particularly for symptom-based classification tasks. The findings suggest that LLMs offer a more intuitive and efficient approach compared to traditional neural networks, paving the way for future research in this domain. Future work should focus on expanding the dataset and exploring more sophisticated fine-tuning techniques to further improve the diagnostic accuracy of LLMs.

## Figures and Tables

**Table 1:** Summary of the models employed: GPT-2, BERT, Distill-BERT, and TinyBERT, including their names, numbers of parameters, and descriptions.

Model	Full Name	Number of Parameters	Pros	Cons	Originality	Results
GPT-2	Generative Pre-trained Transformer 2	1.5 billion	High-quality text generation, versatile, large-scale pre-training.	Computationally expensive, requires significant resources for fine-tuning.	Introduced large-scale unsupervised pre-training for generative tasks.	Achieved state-of-the-art performance in text generation tasks.
BERT-Base	Bidirectional Encoder Representations from Transformers	110 million	Strong performance on a wide range of NLP tasks, bidirectional context.	Large model size, slower inference compared to distilled versions.	Pioneered bidirectional pre-training for contextualized word representations.	Set new benchmarks in tasks like question answering and sentiment analysis.
Distill-BERT	Distilled BERT	66 million	Faster inference, reduced resource requirements, retains BERT’s accuracy.	Slight performance drop compared to BERT-Base.	Introduced knowledge distillation to compress BERT while maintaining performance.	Achieved near-BERT performance with significantly fewer parameters.
TinyBERT (4-layer)	Tiny BERT	~14 million	Extremely lightweight, suitable for edge devices, fast inference.	Reduced performance compared to larger models, limited capacity.	Focused on extreme model compression for low-resource environments.	Demonstrated competitive performance in resource-constrained settings.

**Table 2:** The Acute Inflammations dataset attribute descriptions [16].

Attribute	Description	Data Type	Range/Values
Temperature	Body temperature of the patient	Numeric	35–42 °C
Nausea	Presence of nausea	Categorical	Yes, No
Lumbar Pain	Presence of lumbar pain	Categorical	Yes, No
Urine Pushing	Continuous need for urination	Categorical	Yes, No
Micturition Pains	Pain during urination	Categorical	Yes, No
Urethra Inflammation	Inflammation, itching, or swelling of the urethra outlet	Categorical	Yes, No

**Table 3:** LLM performance evaluation.

	GPT-2 (%)	BERT (%)	Distill-BERT (%)	TinyBERT (%)
Accuracy	1.0	1.0	0.94	0.79
Precision	1.0	1.0	1.0	0.75
F1-score	1.0	1.0	0.94	0.69
Recall	1.0	1.0	0.90	0.66

**Table 4:** Results comparison of plain neural networks and LLMs.

Models	Accuracy (%)
ANN	72
1D-CNN	64
GPT-2	100
BERT	100
Distill-BERT	94
TinyBERT	79

**Table 5:** Few-shot learning of DeepSeek Chat.

Metrics	Acute Inflammation	Nephritis
Accuracy	0.72	
Precision	0.875	0.5
Recall	0.875	0.25
F1-score	0.875	0.33

## Data Availability

Data used for training and testing the models are obtained from a publicly available dataset: https://archive.ics.uci.edu/dataset/184/acute+inflammations (accessed on 10 October 2024).
